# Supplementary material for: An open-source framework for large-scale, flexible evaluation of biomedical text mining systems
Source: J Biomed Discov Collab. 2008 Jan 29;3:1. doi: 10.1186/1747-5333-3-1 (PMC2276192; doi:10.1186/1747-5333-3-1)
Supplement: Additional file 1 — Evaluation platform source code. The additional file contains the software evaluation framework discussed in this paper. The most current release can be downloaded from SourceForge.net [1]. See the accompanying README file for instructions on installation and use. Also included in the distribution is a collection of UIMA wrappers for some commonly used BioNLP tools and annotated corpora. [file 1747-5333-3-1-S1.zip › bionlp_uima_repository-1.0.1/docs/api/src-html/edu/uchsc/ccp/util/external/abgene/AbGeneEISystem.html]

```
001    /*
002     * Created on Aug 15, 2005
003     *
004     * TODO To change the template for this generated file go to
005     * Window - Preferences - Java - Code Style - Code Templates
006     */
007    package edu.uchsc.ccp.util.external.abgene;
008    
009    import java.io.BufferedOutputStream;
010    import java.io.BufferedReader;
011    import java.io.FileInputStream;
012    import java.io.FileOutputStream;
013    import java.io.IOException;
014    import java.io.InputStreamReader;
015    import java.io.PrintStream;
016    import java.util.ArrayList;
017    import java.util.List;
018    import java.util.Stack;
019    import java.util.regex.Matcher;
020    import java.util.regex.Pattern;
021    
022    import edu.uchsc.ccp.util.RunExecutable;
023    import edu.uchsc.ccp.util.nlp.GenericTextUtils;
024    import edu.uchsc.ccp.util.nlp.annotation.Annotator;
025    import edu.uchsc.ccp.util.nlp.annotation.InvalidSpanException;
026    import edu.uchsc.ccp.util.nlp.annotation.TextAnnotation;
027    import edu.uchsc.ccp.util.nlp.ei.EIFileBasedSystem;
028    import edu.uchsc.ccp.util.nlp.mention.ClassMentionTypes;
029    
030    /**
031     * 
032     * @author williamb
033     * 
034     * NOTE: this will only run in a LINUX/UNIX environment due to LINUX specific commands in KeXEISystem.createExecFile
035     */
036    
037    public class AbGeneEISystem implements EIFileBasedSystem {
038        private String baseDirectory;
039    
040        private final boolean DEBUG = false;
041    
042        private final String abgeneOutputSuffix = ".fn";
043    
044        private String abgeneInputFile = "";
045    
046        private final String inputFilename = "abgene_input.txt"; // input file used for acceptStringInput
047    
048        private final String execFilename = "abgene_exec.sh";
049    
050        /**
051         * Initialize a new AbGene EI System
052         * 
053         * @param baseDirectory -
054         *            the absolute path to the directory where the KeX exprot function is located - there MUST exist a baseDirectory/work
055         *            directory
056         */
057        public AbGeneEISystem(String baseDirectory) {
058            /* Set the base directory */
059            this.baseDirectory = baseDirectory;
060    
061            createExecFile(baseDirectory + "/" + inputFilename);
062        }
063    
064        /*
065         * Create the script to run AbGene. This script incorporates the specific environmental variables that must be set before calling
066         * "run_abgene.perl"
067         */
068        private void createExecFile(String inputFilename) {
069            this.abgeneInputFile = inputFilename;
070            try {
071                PrintStream execFile = new PrintStream(new BufferedOutputStream(new FileOutputStream(baseDirectory + "/" + execFilename)));
072    
073                // AbGene requires a system variable: LD_LIBRARY_PATH to be set
074                execFile.println("export LD_LIBRARY_PATH=.;");
075    
076                // change to the base directory
077                execFile.println("cd " + baseDirectory);
078    
079                // execute AbGene
080                execFile.println("./run_abgene.perl " + inputFilename + ";");
081    
082                execFile.close();
083    
084                // make execFile executable
085                RunExecutable.run("chmod 755 " + baseDirectory + "/" + execFilename, false);
086    
087            } catch (IOException ioe) {
088                System.err.println("IOException while creating abgene_exec.sh file in KeXEISystem...");
089                ioe.printStackTrace();
090            }
091        }
092    
093        /**
094         * Return the outputFileName and path
095         */
096        public String getOutputFilename() {
097            return abgeneInputFile + abgeneOutputSuffix;
098        }
099    
100        /**
101         * Works as it would when using AbGene from the command line. The input argument is a file where each line represents an individual
102         * document to be tagged with gene names. Each line must take the form of:<br>
103         * 
104         * @@ID DocumentText
105         */
106        public List<TextAnnotation> getEntitiesFromFile(String inputFile) {
107            /* generate the script file */
108             createExecFile(inputFile);
109             
110            /* run AbGene to generate the output file */
111            runAbGene();
112            
113            return parseEntitiesFromFile(inputFile);
114        }
115    
116        /*
117         * Run AbGene on the input file, and generate the output file
118         */
119        private void runAbGene() {
120            if (DEBUG) {
121                System.out.println("running AbGene...");
122                RunExecutable.run(baseDirectory + "/" + execFilename, true);
123                System.out.println(" ...complete.");
124            } else {
125                RunExecutable.run(baseDirectory + "/" + execFilename, false);
126            }
127        }
128    
129        /**
130         * Take a String as input, create a temporary input file, then run AbGene just as you would from the command line. The document ID is
131         * appended to the input string in order to create the expected input format.
132         */
133        public List<TextAnnotation> getEntitiesFromString(String inputText, String inputDocumentID) {
134            /* Create a temporary input file */
135            PrintStream ps = null;
136            try {
137                ps = GenericTextUtils.openPrintStream(baseDirectory + "/" + inputFilename);
138            } catch (IOException e) {
139                // TODO Auto-generated catch block
140                e.printStackTrace();
141            }
142    
143            /* Write the document ID followed by the document text */
144            ps.println("@@" + inputDocumentID);
145            ps.println(inputText);
146    
147            /* Save the temporary input file */
148            ps.close();
149    
150            /* Run KeX to generate the output file */
151            runAbGene();
152            /*
153             * We could delete the temporary input file here, but since it will generally be relatively small, and AbGene will often be called
154             * repeatedly, we have chosen to just overwrite the temporary file each time, thereby conserving the time that would be needed to
155             * delete it after each execution.
156             */
157            
158            return parseEntitiesFromString(inputText, inputDocumentID);
159        }
160    
161        /*
162         * get protein annotations from the original text
163         */
164        private List<TextAnnotation> parseHitsFromAbGeneOutputFile(String taggedDocumentID, String taggedText, String originalDocumentID,
165                String originalText) {
166            List<TextAnnotation> returnHits = new ArrayList<TextAnnotation>();
167            // try {
168            /*
169             * Initialize the Regex that will be used for finding the text thought to be protein names The pattern must match /GENE,
170             * /CONTEXTGENE, /MULTIGENE, /NEWGENE and possibly others
171             */
172            // Pattern genePattern = Pattern.compile(" (^|[^\\s]+)/(M?U?L?T?I?N?E?W?GENE)"); // finds GENE and /MULTIGENE tags
173            Pattern genePattern = Pattern.compile("(^|\\s)([^\\s]+)/([A-Z]*GENE)");
174            Matcher geneMatcher;
175    
176            if (DEBUG) {
177                System.out.println("TAGGED TEXT: [" + taggedDocumentID + "] " + taggedText);
178                System.out.println("ORIGINAL TEXT: [" + originalDocumentID + "] " + originalText);
179            }
180    
181            /*
182             * Saftey check: make sure the document IDs of the tagged text and the original text match before continuing; if they do not, then
183             * error.
184             */
185            if (originalDocumentID.equals(taggedDocumentID)) {
186    
187                /* Search the tagged text for protein names indicated by /GENE or /MULTIGENE tags appended to the words */
188                geneMatcher = genePattern.matcher(taggedText); // had to add space so that matches at beginning of sentence are
189                // caught
190                // int startIndex;
191                // int lastEndIndex = 0;
192    
193                /* geneText will hold the protein name as captured from the regex */
194                String geneText = "";
195    
196                /* isMultiText will hold either "GENE" or "MULTIGENE", as captured from the regex */
197                String isMultiText = "";
198    
199                TextAnnotation currentAnnotation = null;
200    
201                /* foundMulti is set to true is MULTIGENE is seen, false otherwise */
202                boolean foundMulti = false;
203    
204                /* initialize a Stack to hold annotations. We will empty the stack every time we find the end of a MULTIGENE */
205                Stack<TextAnnotation> multigeneAnnotationsStack = new Stack<TextAnnotation>();
206    
207                /*
208                 * In order to ensure we capture duplicate mentions of the same protein name in the text, we must keep track of where we start
209                 * to search for matches in the text. If returnHits is empty, then start looking from zero, else start looking from the end
210                 * index of the last annotation added to the returnHits list
211                 */
212                int startLookingForMatchesAtIndex = 0;
213    
214                /* Initialize an annotator */
215                Annotator abgeneAnnotator = new Annotator(new Integer(4), "", "ABGENE", "Tanabe");
216                
217                while (geneMatcher.find()) {
218                    /* Group 1 is either a space, or the beginning of a line */
219                    /* extract the protein text from the taggedDocument */
220                    geneText = geneMatcher.group(2);
221                    /* extract GENE or MULTIGENE for this protein */
222                    isMultiText = geneMatcher.group(3);
223                    /* set foundMulti = true if it is a MULTIGENE */
224                    foundMulti = isMultiText.equals("MULTIGENE");
225    
226                    List<TextAnnotation> proteinAnnotations = GenericTextUtils.findTargetInText(geneText, originalText, startLookingForMatchesAtIndex, originalDocumentID,
227                            ClassMentionTypes.PROTEIN, abgeneAnnotator, false);
228    
229                    /*
230                     * Here, we only want to deal with the initial proteinAnnotation since we must keep track of whether or not it is a
231                     * MULTIGENE mention
232                     */
233                    if (proteinAnnotations.size() > 0) {
234                        currentAnnotation = proteinAnnotations.get(0);
235    
236                        /* If this is a MULTIGENE, then push it onto the stack */
237                        if (foundMulti) {
238                            multigeneAnnotationsStack.push(currentAnnotation);
239                        } else {
240                            /*
241                             * This is not a MULTIGENE, check the multigeneAnnotationsStack. If there is anything on the stack, pop them off and
242                             * concatenate the annotations, then store the current annotation
243                             */
244                            if (!multigeneAnnotationsStack.isEmpty()) {
245                                returnHits.add(emptyMultigeneAnnotationsStack(multigeneAnnotationsStack));
246                            }
247    
248                            returnHits.add(currentAnnotation);
249                        }
250                    } else {
251                        System.out.println("************  AbGene missed gene: " + geneText + "  *********** + documentID: " + originalDocumentID + " startIndex" + geneMatcher.start());
252                    }
253                    
254                    /* Update startLookingForMatchesAtIndex to be the end of the span of the most recent additiong to the returnHits lise. */
255                    if (returnHits.size() > 0) {
256                        startLookingForMatchesAtIndex = returnHits.get(returnHits.size()-1).getAnnotationSpanEnd();
257                    }
258                } /* End "while (geneMatcher.find())" */
259    
260                /* Make sure there is not a MULTIGENE waiting in the stack */
261                if (!multigeneAnnotationsStack.isEmpty()) {
262                    returnHits.add(emptyMultigeneAnnotationsStack(multigeneAnnotationsStack));
263                }
264    
265            } else { /* the original text and tagged text document IDs to do match, so there must be a problem somewhere. */
266                System.err.println("ERROR, document IDs do not match up: tagged_docID: " + taggedDocumentID + "  orig_docID: "
267                        + originalDocumentID);
268            }
269    
270            // } catch (Exception e) {
271            // System.err.println("Error while parsing KeX output file. KeXEISystem.");
272            // e.printStackTrace();
273            // }
274            return returnHits;
275    
276        }
277    
278        /*
279         * Concatenates text annotations in a stack into a single annotation, but checks to make sure they are contiguous before doing so
280         */
281        private TextAnnotation emptyMultigeneAnnotationsStack(Stack<TextAnnotation> multigeneAnnotationsStack) {
282            TextAnnotation multigeneAnnotation = multigeneAnnotationsStack.pop();
283            while (!multigeneAnnotationsStack.isEmpty()) {
284                /* pop off annotations, check to make sure they are contiguous, then concatenate */
285                TextAnnotation nextMultiPart = multigeneAnnotationsStack.pop();
286                if (nextMultiPart.getAnnotationSpanEnd() == (multigeneAnnotation.getAnnotationSpanStart() - 1)) {
287                    /* then they are contiguous, so concatenate */
288                        multigeneAnnotation.setAnnotationSpanStart(nextMultiPart.getAnnotationSpanStart());
289                    multigeneAnnotation.setCoveredText(nextMultiPart.getCoveredText() + " " + multigeneAnnotation.getCoveredText());
290                }
291            }
292            /* Check to make sure stack is now empty... error if not */
293            if (!multigeneAnnotationsStack.isEmpty()) {
294                System.err
295                        .println("ERROR. MULTIGENE Stack is not empty when exiting emptyMultigeneAnnotationsStack() method in AbGeneEISystem.");
296            }
297            return multigeneAnnotation;
298        }
299    
300        /**
301         * Return an List of TextAnnotations for the document found in the file
302         */
303        private ArrayList<TextAnnotation> parseEntitiesFromFile(String originalTextFilename) {
304            ArrayList<TextAnnotation> returnHits = new ArrayList<TextAnnotation>();
305            try {
306                /* Initialize a file reader for the tagged output file */
307                BufferedReader br = new BufferedReader(new InputStreamReader(new FileInputStream(this.getOutputFilename())));
308                /* Initialize a file reader for the original text input file */
309                BufferedReader br_origText = new BufferedReader(new InputStreamReader(new FileInputStream(originalTextFilename)));
310                String line;
311    
312                String originalDocumentID;
313                String originalDocumentText;
314                String taggedDocumentID = "";
315                String taggedDocumentText;
316    
317                /* ignore the first line of the file as it states something like: "Threshold set to 0!" */
318                br.readLine();
319    
320                /* Prime the system by getting the first document ID */
321                if ((line = br.readLine()) != null) {
322                    taggedDocumentID = line.substring(2, line.indexOf("/"));
323                }
324    
325                while ((line = br.readLine()) != null) {
326                    /*
327                     * tagged documents are on multiple lines due to KeX processing first line is the doc id, the rest are sentences
328                     */
329    
330                    /* get docText from file, remove line breaks within text */
331                    taggedDocumentText = line;
332                    while ((line = br.readLine()) != null && !line.startsWith("@@")) {
333                        taggedDocumentText += (line + " ");
334                    }
335    
336                    /* get original (untagged text) from file so we can determine the span */
337                    originalDocumentID = br_origText.readLine().substring(2).trim();
338                    originalDocumentText = br_origText.readLine();
339    
340                    /* Find the protein annotations in the given document */
341                    List<TextAnnotation> proteinAnnotations = this.parseHitsFromAbGeneOutputFile(taggedDocumentID, taggedDocumentText,
342                            originalDocumentID, originalDocumentText);
343                    returnHits.addAll(proteinAnnotations);
344    
345                    /* get docID for next document */
346                    if (line != null) {
347                        taggedDocumentID = line.substring(2, line.indexOf("/"));
348                    }
349                }
350    
351                /* close file readers */
352                br.close();
353                br_origText.close();
354    
355            } catch (Exception e) {
356                System.err.println("Error running AbGeneEISystem...returnHitsFromFile().");
357                e.printStackTrace();
358            }
359            return returnHits;
360        }
361    
362        private ArrayList<TextAnnotation> parseEntitiesFromString(String originalDocumentText, String originalDocumentID) {
363            ArrayList<TextAnnotation> returnHits = new ArrayList<TextAnnotation>();
364            try {
365                /* Open reader for the AbGene output file */
366                BufferedReader br = new BufferedReader(new InputStreamReader(new FileInputStream(this.getOutputFilename())));
367                String line;
368    
369                String taggedDocumentID = "";
370                String taggedDocumentText;
371    
372                /* ignore the first line of the file as it states something like: "Threshold set to 0!" */
373                br.readLine();
374    
375                /* Prime the system by getting the first document ID */
376                if ((line = br.readLine()) != null) {
377                    taggedDocumentID = line.substring(2, line.indexOf("/"));
378                }
379    
380                while ((line = br.readLine()) != null) {
381                    /* tagged documents are on multiple lines due to KeX processing first line is the doc id, the rest are sentences */
382                    /* get docText from file, remove line breaks within text */
383                    taggedDocumentText = line;
384                    while ((line = br.readLine()) != null && !line.startsWith("@@")) {
385                        taggedDocumentText += (line + " ");
386                    }
387    
388                    /* Find the protein annotations in the given document */
389                    List<TextAnnotation> proteinAnnotations = this.parseHitsFromAbGeneOutputFile(taggedDocumentID, taggedDocumentText,
390                            originalDocumentID, originalDocumentText);
391                    returnHits.addAll(proteinAnnotations);
392    
393                    /* The next line should be null */
394                    if (line != null) {
395                        /* There should only be one document in this file, so ERROR if the line is not null */
396                        taggedDocumentID = line.substring(2, line.indexOf("/"));
397                        System.err.println("WARNING in AbGeneEISystem. Unexpected document found during returnHitsFromString");
398                    }
399                }
400    
401                /* close file reader */
402                br.close();
403    
404            } catch (Exception e) {
405                System.err.println("Error running AbGeneEISystem... returnHitsFromString().");
406                e.printStackTrace();
407            }
408            return returnHits;
409        }
410    
411    }
```
